# Function and Biomarkers of the Blood-Brain Barrier in a Neonatal Germinal Matrix Haemorrhage Model

**DOI:** 10.3390/cells10071677

**Published:** 2021-07-02

**Authors:** Erik Axel Andersson, Eridan Rocha-Ferreira, Henrik Hagberg, Carina Mallard, Carl Joakim Ek

**Affiliations:** 1 Institute of Neuroscience and Physiology, Sahlgrenska Academy, University of Gothenburg, Medicinaregatan 11, 413 90 Gothenburg, Sweden; axel.andersson@gu.se (E.A.A.); carina.mallard@neuro.gu.se (C.M.); 2 Institute of Clinical Sciences, Sahlgrenska Academy, University of Gothenburg, 413 90 Gothenburg, Sweden; eridan.rocha.ferreira@gu.se (E.R.-F.); henrik.hagberg@obgyn.gu.se (H.H.)

**Keywords:** germinal matrix haemorrhage, blood-brain barrier, neonatal brain, tight-junctions, preterm

## Abstract

Germinal matrix haemorrhage (GMH), caused by rupturing blood vessels in the germinal matrix, is a prevalent driver of preterm brain injuries and death. Our group recently developed a model simulating GMH using intrastriatal injections of collagenase in 5-day-old rats, which corresponds to the brain development of human preterm infants. This study aimed to define changes to the blood-brain barrier (BBB) and to evaluate BBB proteins as biomarkers in this GMH model. Regional BBB functions were investigated using blood to brain ^14^C-sucrose uptake as well as using biotinylated BBB tracers. Blood plasma and cerebrospinal fluids were collected at various times after GMH and analysed with ELISA for OCLN and CLDN5. The immunoreactivity of BBB proteins was assessed in brain sections. Tracer experiments showed that GMH produced a defined region surrounding the hematoma where many vessels lost their integrity. This region expanded for at least 6 h following GMH, thereafter resolution of both hematoma and re-establishment of BBB function occurred. The sucrose experiment indicated that regions somewhat more distant to the hematoma also exhibited BBB dysfunction; however, BBB function was normalised within 5 days of GMH. This shows that GMH leads to a temporal dysfunction in the BBB that may be important in pathological processes as well as in connection to therapeutic interventions. We detected an increase of tight-junction proteins in both CSF and plasma after GMH making them potential biomarkers for GMH.

## 1. Introduction

Improved obstetric and neonatal care has contributed to increasing survival rates of extremely preterm infants (defined as infants born before pregnancy week 28). However, these infants have a high risk of suffering from germinal matrix haemorrhage (GMH). The incidence of GMH is around 20% in low-birth-weight (<1500 g) preterms, and as high as 35–40% in very low birth-weight preterms (<750 g), and, thus, is a major cause of morbidity and death in these groups of infants [1]. GMH involves the rupture of blood vessels in the germinal matrix, a specialised brain area during development rich in neurons and glia. GMH occurs mostly in the first few days after birth in preterm infants, with 50% within 6h of birth [2]. The pathophysiology of GMH is still somewhat unclear but is thought to be multifactorial. One hypothesis often put forward is that germinal matrix vessels are more fragile than other vessels of the brain and thus more prone to rupture. There is evidence that completion of the neurovascular unit occurs later in this region, with less coverage of both astrocytic end-feet and pericytes [3,4], suggesting the particular vulnerability of this region. Additionally, regulation of cerebral blood flow (CBF) that is normally tightly controlled by auto-regulatory mechanisms is not fully developed in preterm infants [5]. Thus, shear flow stress on blood vessels from fluctuations of CBF combined with lesser supportive structures of blood vessels in the GM region may be part of the pathogenesis. This is supported by the fact that stabilising the CBF in infants can reduce the risk of GMH [6]. Other risk factors such as hypoxia, hypoglycaemia, and inflammation are yet to be clarified. If the haemorrhage breaks the neuroependyma and then extends into the brain ventricle, it is termed GMH-IVH, which is associated with a worse neurodevelopmental outcome and higher mortality [7]. This, in turn, is often linked to difficulties in cerebrospinal fluid drainage with shunting of cerebrospinal fluid (CSF), which poses its own risks, necessary in order to manage these complications [8]. One specialisation of the cerebrovasculature is its ability to tightly control the inward and outward passage of molecules between blood and brain. This concept is generally known as the blood-brain barrier (BBB), although the term is slightly misnamed since it encompasses not only barrier mechanisms but also specialised transport systems that will enhance entry of some blood solutes such as nutrients into the brain. Still, the blood-brain barrier is best recognised for keeping blood solutes out of the brain, making it difficult to develop effective pharmacotherapies for neurological disorders. There has been a general misconception that the BBB does not exist in the foetus. We and others have shown that indeed the BBB appears to form and become functional as soon as blood vessels penetrate into the brain [9,10,11,12], and the incompletion of the endothelial barrier is not thought to be underlying the pathophysiology of GMH [13]. Although measurements of BBB function are difficult to perform in human foetuses, the molecular make-up of brain blood vessels suggests a mature BBB phenotype in human foetuses [14]. Given that the vasculature is central in the pathophysiology and that BBB function is an important consideration in relation to potential therapies, we sought to determine vascular barrier function in relation to GMH. For this, we used a model of GMH in new-born rats, which we recently characterised, that shows several of the hallmarks of GMH in humans [15]. This model is based on postnatal day 5 rats, which are developmentally similar to preterm human infants born between approximately 26 to 32 weeks of gestation based on neurodevelopmental hallmarks, such as cortical development [16,17]. We used several approaches to determine BBB function following GMH and could see leaking vessels for days after GMH in the vicinity of the hematoma, as well as smaller changes in BBB function in more distant brain areas. In addition, we measured the levels of BBB proteins claudin-5 (CLDN5) and occludin (OCLN) in CSF and plasma following GMH in order to test their potential as biomarkers and found an increase in both CSF and plasma of these proteins.

## 2. Materials and Methods

### 2.1. Animals

Postnatal day 5 (PND5) Wistar rat pups were bred in-house at the Laboratory for Experimental Biomedicine of Gothenburg University (parents were sourced from Janvier Labs, Le Genest-Saint-Isle, France) and maintained under normal housing conditions with a 12 h light/dark cycle and free access to water and standard laboratory fodder. Animals of both sexes and different litters were used for the experiments, and care was taken to minimise the number of animals used and to maintain an even sex balance in all experimental and control groups. All experiments were approved by the Gothenburg Committee of the Swedish Animal Welfare Agency (Application nos. 825-2017) and performed in accordance with the ARRIVE guidelines. A total of 91 animals were used throughout the study, and the number of animals used per experiment is specified in the Methods and Results.

### 2.2. Germinal Matrix Haemorrhage (GMH)

GMH was induced in PND5 rats as previously described; this experimental approach induces brain injury, particularly in striatum, and the surrounding white matter [15]. In summary, isoflurane-anaesthetized (5% induction, 1.5% maintenance) rat pups were slowly injected with collagenase VII (Sigma-Aldrich, Saint-Louis, MO, USA) at a dose of 0.3 U/mL into the medial striatum of the right hemisphere, in proximity to the germinal matrix. Two µL infusions were made with a 27G (0.4 mm) needle connected to a Hamilton syringe fitted in an infusion pump (CMA/100 microinjection pump) set to a flow rate of 1 µL/min. The procedure typically lasted for 2–5 min. After infusions, the animals were allowed to recover on a heating pad set to 35 °C and then returned to their dams. All animals survived the GMH-model and until the end of experimentation.

### 2.3. Functional Blood-Brain Barrier Assessment, Visible Tracers

To assess BBB function in pups after GMH, we performed BBB tracer experiments to detect vascular leakage of tracers at the individual blood vessel level. We used low-molecular-weight biotin-ethylene-diamine (neurobiotin, 286 Da, Vector Laboratories, CA, USA) and a larger biotin-dextran (BDA, 10,000 Da, ThermoFisher, MA, USA). Doses and tracers were decided from our previous studies [10,18]. At various times after GMH (2 h, 6 h, 24 h and 5 days), animals were injected with tracers dissolved in saline; neurobiotin was injected ip at a concentration of 2.0–2.5 mg per animal, and the same dose of BDA was slowly injected retro-orbitally under full anaesthesia. Three animals were used per tracer and time-point. Animals were euthanised with an overdose of pentobarbital at either 20 min (neurobiotin) or at 10 min (BDA) after the injection. The heart was punctured, and the brain immediately dissected out. The brain was placed on a flat surface, and the posterior part of the hemispheres removed with a razor blade, so the hematoma was in the middle of the remaining part of the cortex. The brain was then immediately immersed in cold buffered fixative (Histofix; Histolab, Gothenburg, Sweden) and left for 24 h at 4 °C. The brains were embedded in 4% agarose and 60 µm coronal sections were cut in a Leica 1200 VT vibratome (Leica Biosystems, Wetzlar, Germany) and stored in PBS until analysis.

### 2.4. Visualisation of Tracers

The injected biotinylated tracers were visualised in 60 µm brain sections using 3′-diaminobenzidine (DAB). In between the different steps below, sections were washed with PBS containing 0.25% Triton X-100. In short, residual agarose was removed from the sections before endogenous peroxidase was blocked by incubation with 1% H_2_O_2_ in PBS and incubated overnight with the Vectastain Elite ABC HRP kit (Vector Laboratories). Sections were then incubated with DAB dissolved in a sodium acetate mixture with β-D-glucose, ammonium chloride, nickel sulphate and β-glucose-oxidase before they were mounted in water-based CC/Mount (Sigma-Aldrich, MO, USA). DAB-stained sections were photographed with a BX60 microscope equipped with a TH4-200 light-source using the cellSens software (Olympus, Tokyo, Japan), larger tiled and stitched images of entire sections were acquired with a Zeiss AxioImager Z2 equipped with an MRc AcioCam using the ZEN Blue software (Zeiss, Oberkochen, Germany).

### 2.5. Quantification of Leaking Blood Vessels and Hematoma Size

The size of the hematoma and the number of leaking blood vessels outside the hematoma were quantified in tiled/stitched images of entire DAB-stained brain sections from animals injected with the neurobiotin- and BDA-tracers using the Fiji build of ImageJ [19]. Leaking blood vessels were identified based on the extravasated tracer residue surrounding them. Leaking blood vessels in the sections were marked with the cell counter plugin, and the size of the hematoma was measured by encircling a region of interest around the hematoma. Additionally, the distance from the edge of the hematoma to the furthest leaking vessel was measured in each brain, using two sections per animal (hippocampal and striatal level), and the results averaged. *n* = 3 per time-point.

### 2.6. Functional Blood-Brain Barrier Assessment by Sucrose Extravasation

A more quantitative assessment of regional BBB function was performed by measuring sucrose blood to brain uptake at 2 h, 6 h, 24 h and 5 days after GMH, together with control animals aged 5, 6 and 10 days, as we have previously described [20]. Briefly, ^14^C-labelled sucrose was injected ip at a dose of 2 µCI per animal and animals killed at 30 min after injection with an overdose of pentobarbital. Blood was collected from the heart and plasma separated by centrifugation. Different regions of the left and right brain hemispheres were dissected out, where we specifically separated areas outside the hematoma in the ipsilateral hemisphere as well as areas more distant to hematoma in both hemispheres. Peri-hematoma regions were collected at least one millimetre from the hematoma to ensure no ruptured vessels were included to test BBB function outside the region of cerebrovascular ruptures. One animal was excluded from the study as the bleeding had reached the left hemisphere. Brain tissues and plasma were collected in pre-weighed scintillation vials, dissolved with 0.5 mL Solvable (PerkinElmer, MA, USA) overnight at 37 °C and after visual assertion that tissues were dissolved, 5 mL of Ultima Gold scintillation cocktail (PerkinElmer) was added to each vial. The radioactivity in each vial was determined by liquid scintillation counting using a Tri-Carb 4910TR (PerkinElmer) and calculated as dpm/g tissue and a ratio between brain samples and plasma calculated as a measure of BBB permeability with blood space corrections of brain samples as in a previous study [21]. *n* = 7 (control groups) or 6 (GMH groups) for all time-points.

### 2.7. Fluorescent Immunohistochemistry and 3D Imaging

For fluorescent immunohistochemistry, agarose was removed, and the sections were permeabilised in PBS/0.25% Triton X-100 for 3 h before blocking in 4% donkey serum for 1 h at RT, followed by incubation with primary antibody mouse-anti-rat CLDN5 (ThermoFisher, 4C3C2, diluted 1/250 in PBS) for two nights at 4 °C. Followed by incubation with AlexaFluor donkey-anti-mouse 488 (Invitrogen, MA, USA, diluted 1/250) and streptavidin-conjugated Texas Red (Vector Laboratories) O/N at 4 °C. Sections were mounted in ProLong Gold Antifade (ThermoFisher). Z-stacks of blood vessels in fluorescent sections were acquired with a Zeiss LSM 800 confocal microscope and 3D-imaged in ZEN blue.

### 2.8. Levels of Tight-Junction Proteins in CSF and Plasma after GMH

For all time-points after GMH and time-matched control (2 h, 6 h, 24 h and 5 days), animals were euthanised with a lethal overdose of pentobarbital. Blood was collected by cardiac puncture with EDTA-treated syringes and centrifuged at 2000× g for five min to separate the plasma. Cerebrospinal fluid was collected via glass capillaries from the cisterna magma and was checked for blood contamination [21]; no contamination was detected in any sample. Samples were placed on dry ice directly after collection and later long-term stored at −80 °C before analysis. The levels of tight-junction proteins were measured in plasma and CSF from GMH and control animals using commercially available ELISA kits for OCLN (Cusabio, Wuhan, China) and CLDN5 (Nordic BioSite, Stockholm, Sweden) following the manufacturer’s instructions. Diluted samples and a series of standards in duplicates were incubated on antibody-coated plates before stepwise incubation with a secondary antibody, horseradish peroxidase-avidin, a 3,3′,5,5′-tetramethylbenzidine substrate and the stop-solution. The optical density was determined in a Spectramax Plus microplate reader (San Jose, CA, USA) set to 450 nm with 540 nm wavelength-correction (OCLN) or 450 nm (CLDN5) and the protein concentration was calculated from the standard curve. CSF was diluted 10 times and plasma 20 times, and samples from the same animals were analysed for both proteins, *n* = 6 (control groups) or 7 (GMH-groups) for all time-points.

### 2.9. Statistics and Graph

Statistical analyses were made using GraphPad Prism version 9.00 for Windows (GraphPad Software, CA, USA). Analyses include one-way ANOVA with Dunnett’s multiple comparisons test or the Holm–Šídák multiple comparison tests as well as paired and unpaired *t-*tests. Levels of tight-junctional proteins between sexes were analysed by two group comparisons adjusting for time as a factor (Qlucore Software). Figures were designed in Affinity Photo and Designer (Serif Europe, West Bridgford, United Kingdom). Data are presented as mean ± SD.

## 3. Results

### 3.1. Functional Blood-Brain Barrier Assessment Using Molecular Tracers

**Figure 1:**
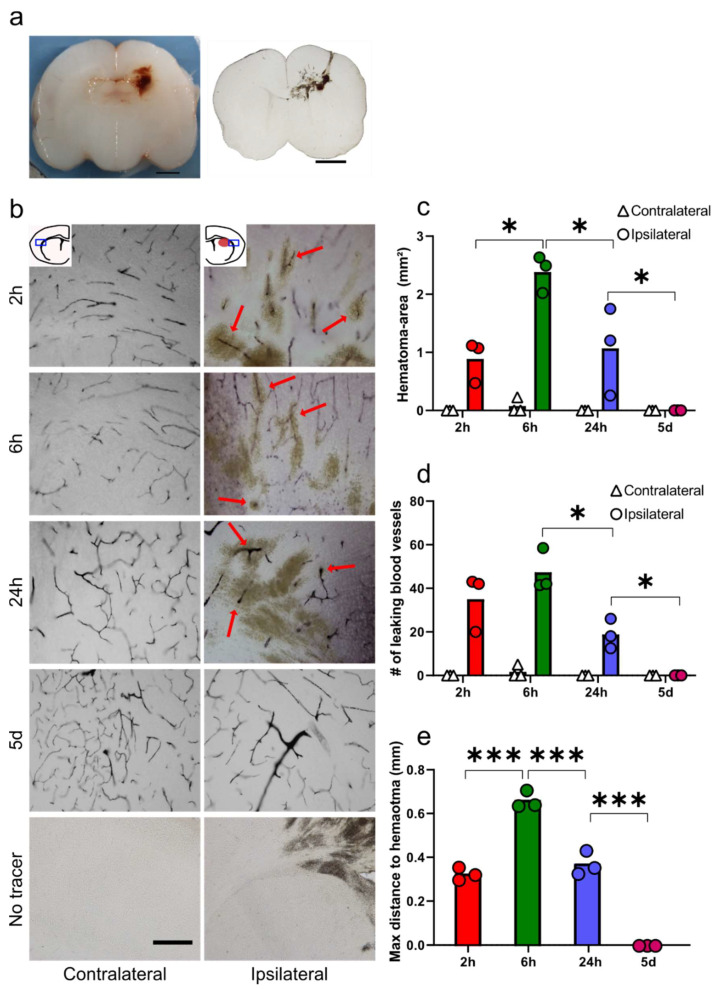
Functional blood-brain barrier assessment and quantification of leaking blood vessels using molecular tracers. (**a**) The hematoma is macroscopically visible in brains during processing and after sectioning. (**b**) Representative images of tracer distribution (red arrows) found in the area around the hematoma at different times following germinal matrix haemorrhage (GMH). Note that around the core hematoma, blood-brain barrier (BBB) function was compromised in some vessels up until 24 h following GMH. (**c**) The size of the hematoma after GMH. (**d**) Number of leaking blood vessels at different times after GMH in the peri-hematoma region. (**e**) Maximum distance to leaky blood vessels from the edge of the hematoma animals after GMH. One-way ANOVA with the Holm–Šídák multiple comparisons test, * *p* ≤ 0.05, *** *p* ≤ 0.001. *n* = 3 for all time-points, bar graphs represent mean values. Scale bars are 2 mm (**a**) and 100 µm (**b**).

Collagenase injection in the medial striatum gives rise to GMH-like bleeding around the injection site. The bleed was macroscopically visible before and after vibratome-sectioning (Figure 1a). To measure characteristics of the hematoma, entire 60 µm brain-section sections from animals injected with tracer-molecules BDA or neurobiotin at different time-points after GMH were DAB-visualised and imaged in larger tiled and stitched images. The number of tracer-leaking blood vessels was quantified and compared in both hemispheres. Figure 1b shows representative images from both hemispheres at all time-points with leaking blood vessels marked with arrows. Both tracers gave similar staining, and both neurobiotin (2 and 6 h) and BDA (24 h and five days) are shown. All tracer-stained blood residues were manually delineated, and the contralateral hemisphere compared to the ipsilateral hemisphere (Figure 1c). The average size of the hematoma in the ipsilateral hemisphere was 0.89 ± 0.29 mm^2^ at 2 h, with an increase to 2.38 ± 0.26 mm^2^ at 6 h (*p* = 0.010), 18 h later the hematoma was reduced to 1.07 ± 0.61 mm^2^ (*p* = 0.014). By five days after GMH, no hematoma was visible in any section (*p* = 0.019 compared to 24 h). The number of leaking blood vessels was 35 ± 11 in the ipsilateral hemisphere 2 h after GMH, and 47 ± 8 at 6 h, which was reduced to 19 ± 5 by 24 h (*p* = 0.0.012), while no leaking vessel could be seen at five days post-GMH, *p* = 0.039 compared to 24 h (Figure 1d). No hematoma or leaking vessels were observed either in control animals or in the contralateral hemispheres at 2 h, 6 h, 24 h or five days after GMH, except in one animal at 6 h after GMH where the bleed had infiltrated to the border of the contralateral hemisphere. Sections from animals that had undergone GMH but not injected with the tracer showed no labelling. The average distance from the edge of the hematoma to the most distant leaking vessel was 0.33 ± 0.02 mm at 2 h, which increased to 0.67 ± 0.03 mm at 6 h (*p* ≤ 0.001), followed by a decrease to 0.37 ± 0.05 mm at 24 h (*p* ≤ 0.001) (Figure 1e). *n* = 3 per time-points.

### 3.2. Regional Sucrose Permeability Across Blood-Brain Barrier after GMH

**Figure 2:**
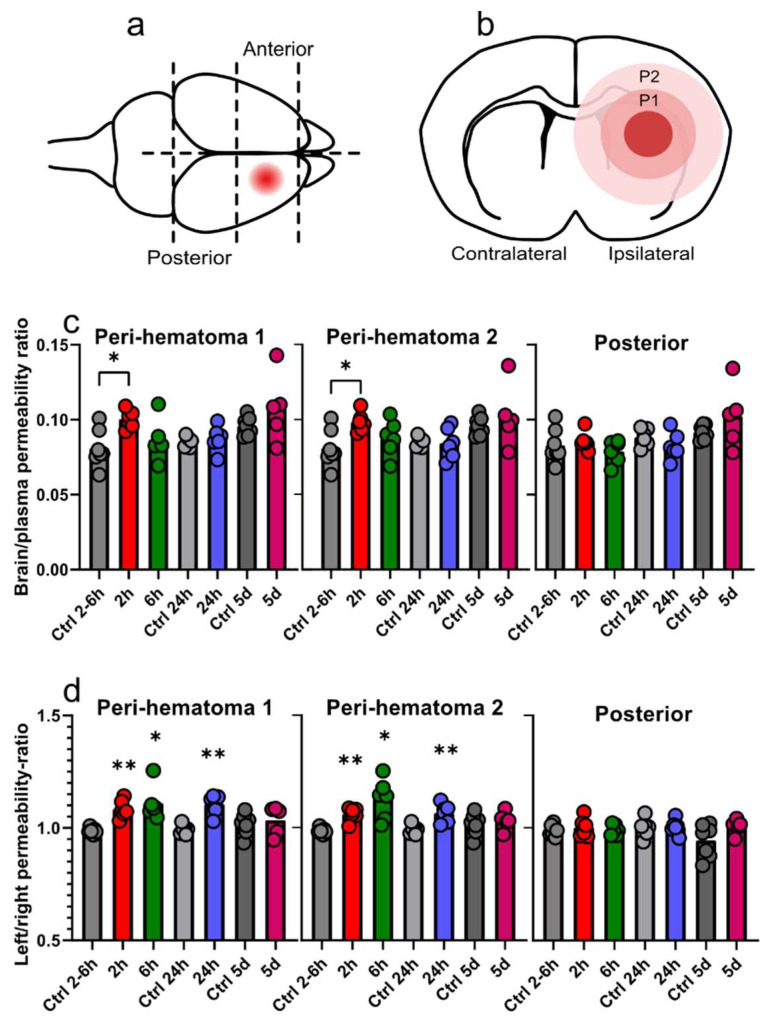
GMH induces spatial and temporal changes in BBB function. Regional BBB function was determined by sucrose permeability at different times after GMH. The regions of measurements are shown in (**a**) sagittal and (**b**) coronal (P1 = peri-hematoma 1, P2 = peri-hematoma 2). (**c**) The sucrose concentration ratios in the ipsilateral regions at 2 h, 6 h, 24 h and 5 days after GMH along with age-matched control animals. (**d**) The sucrose permeability concentration ratios between the ipsilateral and contralateral sides of the brain within animals. Unpaired *t*-tests and one-way ANOVA with Dunnett’s multiple comparison test (**c**), paired *t*-tests (**d**), * *p* ≤ 0.05, ** *p* ≤ 0.01. *n* = 7 (control groups) or 6 (GMH groups) per time-points, bar graphs represent mean values.

The effects of GMH on the BBB was further quantified by measuring the permeability of ^14^C-sucrose. To achieve regional measurements, the brain was dissected into four parts (Figure 2a), and the anterior part of the ipsilateral hemisphere of GMH-animals was further divided into two parts termed peri-hematoma 1 and 2 (Figure 2b). Since blood flow is likely to be compromised in the hematoma, we did not perform analysis in this area and focused on regions around the hematoma where we did not detect leaking vessels from tracer studies. For control animals, the brain was divided into two posterior (ipsi/contra) and two anterior parts (ipsi/contra). A significant increase in BBB permeability was detected 2 h after GMH in both peri-hematoma area 1 (GMH 0.10 ± 0.005 vs. controls 0.081 ± 0.011; *p* = 0.012) and peri-hematoma area 2 (GMH 0.098 ± 0.006 vs. controls 0.081 ± 0.01; *p* = 0.019). When comparing the posterior part of the hemispheres, no differences were found between control and GMH-animals (Figure 2c). We also calculated sucrose permeability ratios within each animal between the ipsi- and contra-lateral sides of the brain. Higher BBB permeability ratios were found at 2 h (1.08 ± 0.03, *p* = 0.002), 6 h (1.11 ± 0.07, *p* = 0.025) and 24 h (1.11 ± 0.04, *p* = 0.001) after GMH in peri-hematoma area 1 and at 2 h (1.06 ± 0.03, *p* = 0.004), 6 h (1.13 ± 0.08, *p* = 0.018), and 24 h (1.07 ± 0.03, *p* = 0.001) in peri-hematoma area 2. These ratios did not differ at 5 days after GMH, neither in the posterior part of hemispheres at any time-point in GMH animals nor in naïve control animals (Figure 2d, *n* = 7 (control groups) or 6 (GMH groups) for all time-points.

### 3.3. 3D Imaging of Leaking and Uninjured Brain Blood Vessels

**Figure 3:**
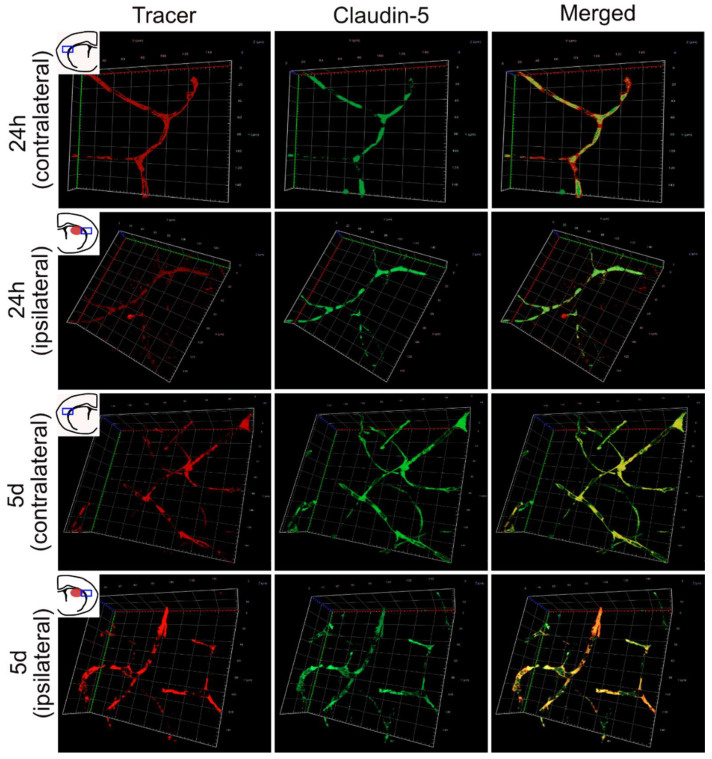
Three-dimensional imaging of tracer and CLDN5 in brains following GMH. Representative images of 3D-imaged z-stacks of vessels immunolabelled for claudin-5 (CLDN5) and tracer molecule taken in the peri-hematoma region of the ipsilateral hemisphere or the corresponding area in the contralateral hemisphere. At 24 h the CLDN5 labelling was discontinuous in vessels that also showed lost barrier integrity (tracer not contained within the vessel) in the ipsilateral hemisphere, while at five days, barrier integrity was re-established in blood vessels with continuous CLDN5 labelling and tracer contained within vessels. In the contralateral side of the brain, CLDN5 immunolabelling always remained continuous, and tracer remained within blood vessels. The x- and y-axes are 140 µm.

Blood vessels adjacent to the GMH-injury were further examined by 3D-imaging of entire vessels from z-stacks acquired by confocal microscopy of thick sections stained for CLDN5 and tracer. Images were taken in the peri-hematoma region and the corresponding area in the contralateral hemisphere. Vessels in the contralateral hemisphere 24 h after GMH showed coherent stretches of stained CLDN5 and tracer throughout the entire sections no injured vessels could be detected (Figure 3). In contrast, vessels that showed extravasation of tracer into adjacent brain parenchyma also showed discontinuous labelling of CLDN5. Five days after GMH, no leaking vessels could be detected in either hemisphere.

### 3.4. BBB Proteins Are Increased in Plasma and CSF after GMH

**Figure 4:**
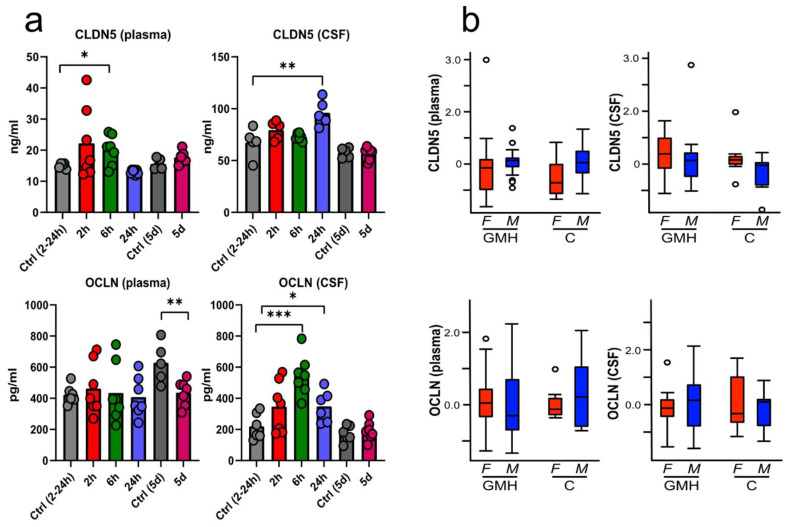
Time-dependent changes of tight junction protein levels in blood plasma and cerebrospinal fluid after GMH. The levels of CLDN5 and occludin (OCLN) was measured using ELISA at 2 h, 6 h, 24 h and five days after GMH, as well as time-matched control animals (**a**). Sex effect of CLDN5 and OCLN animals were investigated by pooling all samples from GMH and control (C) animals and adjusting levels for time. No difference was seen between male (M) and female (F) pups (**b**). Data was analysed by unpaired *t*-tests and one-way ANOVA with Dunnett’s multiple comparison test, * *p* ≤ 0.05, ** *p* ≤ 0.01, and *** *p* ≤ 0.001. *n* = 6 (control groups) or 7 (GMH-groups) for all time-points. Bar graphs represent mean values; box-plots showing median, quartiles, range (whiskers) and outliers (open circles, 1.5* quartile).

Tight-junction proteins were determined at 2 h, 6 h, 24 h and 5 days after GMH in blood plasma and CSF with ELISA-kits for CLDN5 and OCLN and compared to time-matched control groups (Figure 4a). CLDN5-levels in plasma were in the range of 10–20 ng/mL for most samples, with an increase 6 h after GMH (20.1 ± 4.4) compared to controls (14.9 ± 0.7, *p* = 0.04). The protein was more abundant in CSF, with normal levels ranging between 45 and 80 ng/mL, and was higher in GMH animals (96.0 ± 11.3) than controls (67.4 ± 12.3 at 24 h; *p* = 0.009). OCLN-levels in plasma were lower in GMH animals (482.4 ± 141.6) compared to controls (628.7 ± 116.0) at 5 d (*p* = 0.009). Several animals had elevated OCLN in CSF only 2 h after GMH, with significant increases at 6 h (547.6 ± 121.4, *p* ≤ 0.001) and 24 h post GMH (347.9 ± 92.1, *p* = 0.039) compared to controls (220.5 ± 76.9). CLDN5- and OCLN-levels were not different between GMH and control animals in either plasma or CSF, apart from higher levels in the five-day control group (628.7 ± 115.9, *p* = 0.010) for OCLN in plasma. No sex differences were found in the levels of circulating OCLN or CLDN5 at any time-point in neither plasma nor CSF (Figure 4b, *p*-values ranged from 0.21–0.86). See Supplementary Table S1 for average tight-junction proteins concentrations by sex in CSF and plasma. *n* = 6 (control groups) or 7 (GMH-groups) for all time-points.

## 4. Discussion

GMH is a serious cause of death and disability, especially in low birth-weight premature infants, and there is a lack of treatments and diagnostic tools. Our group recently developed a rodent model for grade III and IV GMH using PND5 rats [15] to mimic this condition in the human preterm brain [16]. We had two objectives with this study. Firstly, we wanted to define regional changes to barrier function in the cerebrovasculature following GMH, as this is important both to understand the underlying pathophysiology and to aid potential therapeutic interventions that need to reach brain cells to be effective. Secondly, as BBB proteins have been shown to be potential biomarkers for brain vascular dysfunction in different settings of brain injury, we wanted to determine their levels in both CSF and plasma following GMH. We injected molecular tracers at different times after GMH and visualised these in the brain tissue together with the distribution of the key BBB transmembrane protein CLDN5 in brain blood vessels. The haemorrhage occurred rather quickly after collagenase injections and was visible in the pups within only 15–30 min following the injections. After 2 h, a hematoma was visible in brain sections, and the vascular integrity within the hematoma appeared mostly devoid, although assessment of integrity at the individual blood vessel level is hampered by the hematoma and tracer distribution. In the area surrounding the hematoma, we found a zone up to around 300–350 µm where blood vessels show heterogeneous BBB function with some vessels that remain BBB competent while others have lost their barrier integrity with tracers leaking out quite freely. Outside this zone, all vessels retained tracers. There is a hematoma expansion phase up to at least 6 h, accompanied by a greater number of vessels that lose BBB integrity even further from the hematoma. High-resolution imaging of these vessels in the peri-hematomal region showed that vessels leaking tracer also had lost CLDN5. At 24 h after GMH, the hematoma has shrunk by 45%, and the number of leaky vessels is 40% less compared to 6 h after GMH, consistent with an earlier observation that erythrocytes and iron are cleared by this time [15,16]. There was also a marked reduction of the area with leaky vessels in the peri-hematomal region between 6 and 24 h. These data indicate vascular repair mechanisms starting to re-establish vascular integrity, resulting in a lesser number of leaky vessels and shrinkage of the hematoma by 24 h. This repair phase continues with complete resolution of the hematoma by 5 days, and tracers are all visibly contained within the cerebral vasculature, clearly showing that vascular barrier function is restored. This is further supported by high-resolution 3D imaging of blood vessels showing continuous CLDN5 labelling and tracers retained within the vessels. The short duration between tracer injection and brain collection ensured that the observed tracer extravasation did really come from locally damaged vessels and had not diffused from other parts of the injury [18]. The small discrepancy between tracer studies and sucrose experiments on BBB function in the more distant peri-hematomal regions can most likely be explained by the higher sensitivity in the detection of sucrose in the parenchyma, which only pointed to a modest change in BBB function. The power of tracers is that we can assess BBB integrity at the blood vessel level that is not possible by measuring sucrose flux into the brain; thereby, these techniques complement each other. We previously showed that vascular integrity is lost in the hematomal region after collagenase injections, with many vessels showing fractured staining for both CLDN5 and laminin [15], but did not follow the temporal or spatial changes. Zhang and colleagues [22], who have used a similar GMH model to us in rats, albeit at P7, did measure increased extravasation of Evans blue after GMH, as well as detecting lower levels of occludin, ZO-1 and claudin-3 at 3 days after GMH. Their measurements were performed in whole hemispheres, so it is difficult to judge where vascular integrity was lost or specifically where the loss of these proteins occurred. It should also be mentioned that it has later been established that claudin-3 does not confer BBB properties to cerebral blood vessels, where claudin-5 is dominant, but its main sealing function is in epithelia [23]. They did see neuroprotection by both insulin-like growth factor 1 and fingolimod in this model with a reduction in BBB dysfunction showing that therapies directed towards the stabilisation of the cerebrovasculature could be beneficial in GMH [22,24] and supporting the idea that secondary changes to vascular integrity are a driver of pathogenesis.

The haemorrhage results in the deposition of blood products, as well as giving rise to metabolites that could be toxic and drivers of secondary injury mechanisms. Some of these possible toxic compounds are thrombin, heme, iron and complement factors and pro-inflammatory compounds. For instance, Gao and colleagues used an IVH model in rats, where blood was directly injected into the lateral ventricles, showing that simultaneous thrombin injections resulted in hydrocephalus and increased levels of albumin in peri-ventricular areas indicative of loss of BBB function [25]. Ley and colleagues have made a series of studies using glycerol-induced IVH in rabbits that indicate that extracellular haemoglobin and its ferric state, methaemoglobin, are damaging and pro-inflammatory, and thus a central player in the secondary injury mechanisms [26,27,28]. The development of secondary injury is also driven by neuroinflammation as GMH leads to increases in inflammatory cytokines, microgliosis [29,30] and astrogliosis [15]. Since several of these secondary injury mechanisms could potentially also be damaging to endothelial functions or BBB modulatory, we wanted to further test BBB function in regions more distal to the hematoma. To achieve this, we used radio-tagged sucrose, which can be measured highly accurately in tissues to accurately quantify the BBB permeability [31]. This showed that we could detect a small increase in BBB permeability between GMH and control animals at 2 h after the GMH in peri-hematomal regions, but at later times no change was detected. To increase the resolution of these measurements, we also calculated permeability ratios within each animal where the collagenase-injected hemisphere was compared to the contralateral hemisphere. These ratios are consistently higher in the peri-hematomal regions between 2 and 24 h compared to contralateral areas, indicating an increase in BBB permeability, albeit the magnitude of change is small. The peri-infarct areas where we saw increases in BBB-permeability is also the same areas where the white- and grey-matter tissue loss are most pronounced in this model [15]. The ratios, however, are probably somewhat of an underestimate since oedema is likely occurring predominantly in the haemorrhagic hemisphere, diluting extracellular markers. In addition, ratios within animals should be interpreted with some caution since it is possible that the contralateral hemisphere is somewhat affected in this model. However, overall, our data do not suggest that BBB function is affected in the contralateral hemisphere of GMH animals as no change was detected in this hemisphere from control animals and, furthermore, in GMH animals, the ratios between hemispheres are equal across the uninjured posterior part of the hemispheres. A study using an adult model of brain haemorrhage, where blood was injected into a similar region of the brain to our model, was also not able to detect changes in BBB function in the contralateral hemisphere [32]. Similar to our tracer studies, the sucrose experiments indicated a normalisation of BBB function at 5 days after the haemorrhage.

We and others have shown that elevated levels of tight-junction proteins can be detected in the circulation in rodent models for neonatal hypoxia/ischemia (HI) [20] and adult middle-cerebral artery occlusion [33], as well as in adult human patients with stroke [34] and intracranial haemorrhage [35], making these proteins promising biomarkers for brain vascular dysfunction in different settings of brain injury. Furthermore, the levels of these proteins correlated with the brain injury in neonatal HI [20], while in stroke patients, levels predicted haemorrhagic transformation [34]. We, therefore, wanted to measure these proteins following GMH, where the cerebral vasculature is a central component of the injury process. After GMH, changed levels were detected in both plasma and CSF with the greatest increase in CLDN5 at 6 h in plasma and 24 h in CSF compared to control animals, and elevated OCLN in CSF at 6 h and 24 h, concurring with our previous results from neonatal HI. Our previous results showed that the levels of circulating OCLN in plasma and CLDN5 in CSF are differentially changed by sex, with higher levels in males, which may correspond to that in models for neonatal HI results in more severe injuries in male animals [36,37]. In GMH, we found no sex-dependent influence in tight junction-protein levels in CSF nor plasma. While male infants seem to have a higher risk for developing GMH [38] and get a worse neurological outcome than females [39,40], there are, to our knowledge, no studies that investigate sex-dependency on injury in animal models of GMH. Thus, we can conclude that these proteins potentially could be used as biomarkers in the setting of GMH; however, they need to be further tested in other models of GMH, as well as in clinical samples, in order to evaluate their usefulness in general.

In conclusion, this study shows that the PND5 model of GMH gives rise to a core of haemorrhagic vessels and to a quite defined surrounding region where some vessels lose their integrity, and larger molecules appear to freely move out of vessels. Both these regions expand in the first few hours after the induction of GMH, along with an increased number of leaking vessels. However, rather soon, there is re-establishment of barrier function in blood vessels, with a 50% reduction in the number of leaking vessels at 24 h, and by five days, only vessels with competent BBB function are present. The vasculature is also affected around the haemorrhagic region with a moderate increase in BBB permeability, which seems to normalise at five days after GMH. We can also report that GMH gives rise to increased levels of tight-junction proteins in both CSF and plasma, making them potential biomarkers for GMH damage that should be further evaluated.

## Data Availability

The data behind the conclusions of this study are available from the corresponding author upon reasonable request.

## Supplementary Materials

The following are available online at https://www.mdpi.com/article/10.3390/cells10071677/s1, Table S1: Average tight-junction concentrations separated for group and sex. OCLN and CLDN5 was measured in the blood and plasma of all animals at 2 h, (3m, 4f) 6 h (4m, 3f), 24 h (3m, 4f), 5 d (4m, 3f), 2-24 h controls (3m, 4f), and 5 d controls (3m, 4f). m = male, f = female.
