# Supplementary material for: Function and Biomarkers of the Blood-Brain Barrier in a Neonatal Germinal Matrix Haemorrhage Model
Source: Cells. 2021 Jul 2;10(7):1677. doi: 10.3390/cells10071677 (PMC8303246; doi:10.3390/cells10071677)
Supplement: Supplementary file 1 [file cells-10-01677-s001.zip › cells-1267019-supplementary.pdf]

Table S1. Average tight-junction concentrations separated for group and sex. OCLN and CLDN5 was measured in the blood and plasma of all animals at 2h, (3m, 4f) 6h (4m, 3f), 24h (3m, 4f), 5d (4m, 3f), 2-24h controls (3m, 3f), and 5d controls (3m, 3f). m = male, f = female.

**[TJ-protein]**	**GMH**			**Controls**		
	Males	Females	p-value	Males	Females	*p*-value
CLDN5 (plasma)	17.56 ± 5.26	19.49 ± 8.73	0.8575	15.65 ± 1.41	15.03 ± 1.05	0.3563
OCLN (plasma)	422.56 ± 141.00	458.90 ± 130.88	0.6739	534.24 ± 152.31	502.34 ± 116.33	0.4100
CLDN5 (CSF)	73.87 ± 16.15	75.47 ± 15.54	0.6761	59.31 ± 9.80	65.81 ± 10.04	0.2063
OCLN (CSF)	364.18 ± 185.00	349.25 ± 142.70	0.3485	188.06 ± 39.28	202.82 ± 82.40	0.5824
